# Unclassified Chromosomal Abnormalities as an Indicator of Genomic Damage in Survivors of Hodgkin’s Lymphoma

**DOI:** 10.3390/cancers17152437

**Published:** 2025-07-23

**Authors:** Sandra Ramos, Bertha Molina, María del Pilar Navarrete-Meneses, David E. Cervantes-Barragan, Valentín Lozano, Sara Frias

**Affiliations:** 1 Laboratorio de Citogenética, Instituto Nacional de Pediatría, Ciudad de Mexico 04530, Mexico; sramosa@pediatria.gob.mx (S.R.); bertha_molina@ciencias.unam.mx (B.M.); 2 Laboratorio de Genética y Cáncer, Instituto Nacional de Pediatría, Ciudad de Mexico 04530, Mexico; mnavarretem@pediatria.gob.mx; 3 Departamento de Genética, Hospital Central Sur Alta Especialidad, PEMEX, Ciudad de Mexico 14140, Mexico; david.eduardo.cervantes@pemex.com; 4 Departamento de Hematología, Instituto Nacional de Cancerología, Ciudad de Mexico 14080, Mexico; vlozanoz@live.com.mx; 5 Departamento de Medicina Genómica y Toxicología Ambiental, Instituto de Investigaciones Biomédicas, Universidad Nacional Autónoma de México, Ciudad de Mexico 04510, Mexico

**Keywords:** unclassified chromosomal aberrations, genomic instability, Hodgkin’s lymphoma, cancer survivors, genotoxicity, chemotherapy, radiotherapy

## Abstract

**Background/Objectives**: Hodgkin’s lymphoma (HL) affects 2–4 individuals per 100,000 annually. Standard treatment includes radiotherapy and ABVD chemotherapy, achieving a 95% survival rate. However, HL survivors face an elevated risk of treatment-related morbidity, particularly the development of secondary malignancies. Previous studies have demonstrated that ABVD treatment induces a high frequency of chromosomal aberrations (CAs) in lymphocytes from HL patients, with higher frequencies one year after treatment than during treatment. This study aimed to determine whether HL treatment also induces unclassified chromosomal/nuclear aberrations (UnCAs) in the lymphocytes of HL patients, and whether these alterations may serve as complementary indicators of genomic instability. **Methods**: Peripheral blood lymphocytes from HL patients were collected at three time points: before treatment (BT), during treatment (DT), and one year after treatment (1yAT) with ABVD chemotherapy and radiotherapy. A minimum of 3000 nuclei were analyzed per patient to identify and quantify UnCAs. These results were compared to UnCA frequencies in healthy individuals. **Results**: The percentage of cells presenting UnCAs per 3000 nuclei was 23.92% BT, 18.58% DT, and 30.62% 1yAT. All values were significantly higher (*p* < 0.016) than the 8.16% observed in healthy controls. The increase was primarily driven by free chromatin and micronuclei clusters. UnCA frequency was lower during treatment than one year after, likely due to the elimination of highly damaged cells through apoptosis or lack of proliferative capacity. Over time, however, persistent genomic damage appears to accumulate in surviving cells, becoming more evident post-treatment. A parallel trend was observed between the frequencies of UnCAs free chromatin, micronucleus and micronuclei clusters, and classical CAs, showing a similar pattern of genomic damage induced by therapy. **Conclusions**: The post-treatment increase in UnCAs indicates ongoing genomic instability, possibly driven by the selective survival of hematopoietic stem cells with higher genomic fitness. Given their persistence and association with therapy-induced damage, free chromatin and micronuclei clusters may serve as early biomarkers for secondary cancer risk in HL survivors.

## Simple Summary

This study examined unclassified chromosomal abnormalities (UnCA) in patients with Hodgkin’s lymphoma (HL) before, during, and after anticancer treatment. HL is a rare cancer affecting 2–4 people per 100,000 individuals annually. Although treatment is highly effective, survivors face an increased risk of developing other cancers. In this study, we analyzed blood samples from HL patients and survivors at different stages of treatment, focusing on changes in their chromosomes that do not fit standard categories. These changes include alterations in chromosomes and nuclei. We found that UnCA levels were higher in HL patients than in healthy individuals at all stages; the general UnCA frequency decreased during treatment but increased again one year after treatment, at the expense of two specific UnCAs, free chromatin and micronuclei clusters. Given their persistence and association with genomic instability, these UnCAs might serve as early indicators of secondary cancers in HL survivors.

## 1. Introduction

Hodgkin’s lymphoma (HL) is one of the most common pediatric cancers, accounting for approximately 4.9% of cases in children under 15 years of age [1]. HL is a hematological malignancy with distinct characteristics, including its frequent occurrence in young adults and the presence of rare malignant Reed–Sternberg cells—multinucleated, B-lymphocyte-derived cells—within a microenvironment rich in immune effector cells [2]. The global incidence rate of HL is approximately 1.2 per 100,000 individuals, with higher rates observed in Western Europe (3.0 per 100,000) [3]. Notably, HL has one of the highest survival rates among cancers, with a 5-year survival exceeding 90% following treatment [4,5]. The treatment is based on the administration of chemotherapy, mainly using ABVD (Adriamycin, Bleomycin, Vinblastine, and Dacarbazine) alone or in association with radiotherapy [5,6]. Chemotherapeutic schemes are not specific for neoplastic cells; so, there may be cell damage and genotoxicity in normal tissues, indeed chromosomal instability has been reported in HL survivors treated with ABVD chemotherapy, represented as non-clonal chromosomal aberrations (NCCAs), both numerical and structural [7,8]. Chromosomal instability (CIN) plays a critical role in the generation of novel genomes and karyotypes, leading to a cell population with extensive genomic heterogeneity, an ideal substrate for malignant transformation [9]. Over the long term, this genomic instability can contribute to the development of second malignant neoplasms (SMNs). SMNs are a significant cause of mortality among HL survivors [10]. The incidence of SMNs is estimated to be approximately 14–17% at 20 years post-treatment, increasing to over 20% at 30 years following initial therapy for the primary tumor [4]. This elevated risk is closely associated with the presence of NCCAs in survivors, as these chromosome aberrations (CAs) are indicative of CIN, a widely recognized hallmark of cancer cells. NCCAs contribute significantly to the dynamic nature of the genome during tumorigenesis, with each aberration offering a distinct evolutionary potential. This genomic variability serves as the foundation for the macroevolutionary phase of cancer progression, enabling the emergence of more aggressive or resistant clones [11].

Genome rearrangements resulting from CIN are not confined to the mitotic phase—where chromosomal abnormalities are most commonly observed—but can also arise during other stages of the cell cycle. In this context, additional forms of genomic alterations, referred to as chromosomal/nuclear unclassified chromosomal alterations (UnCAs), have been identified. Although long regarded as cytogenetic artifacts, UnCAs have gained recognition following studies analyzing the responses of oncological cell lines to antineoplastic agents [12]. These investigations have characterized distinct types of UnCAs, which are now considered manifestations of stochastic genomic alterations (NCCAs)**.** Their presence is increasingly recognized as an indicator of genomic instability [13,14]. Heng et al. [15] identified a range of chromosomal/nuclear UnCAs and demonstrated a significant increase in these aberrations in both experimental and clinical samples exposed to genotoxic agents. The identified UnCAs included free chromatin, defective mitotic figures (DMFs), sticky chromosomes, unit fibers, chromosome fragmentation (C-Frag), micronuclei (MN), micronuclei clusters (MN clusters), and abnormal nuclear morphology. Although some of these chromosomal/nuclear UnCAs are extremely damaged genomes, such as chromosomal fragmentation or MN clusters, some of them may retain the ability to survive stress and maintain proliferative capacity. The progeny of these cells, carrying chaotic genomes, significantly contribute to karyotypic heterogeneity, which serves as the substrate for cellular macroevolution toward cancer [12,15,16,17].

Despite their potential relevance, chromosomal/nuclear UnCAs remain poorly characterized. To date, only micronuclei (MN) have been extensively studied and validated as reliable indicators of DNA damage and mitotic defects [18,19,20]. In contrast, other types of UnCAs lack a comparable body of research. Therefore, it is essential to conduct studies that establish baseline frequencies in healthy individuals and assess how these NCCA behave under genotoxic stress. Cancer survivors represent a highly relevant study population due to the lasting side effects that treatment-induced stress can have on their non-cancerous cells. Additionally, studying these patients offers a unique opportunity to explore the in vivo generation of UnCAs as a response to therapeutic stress.

The aim of this longitudinal study is to determine the frequencies of chromosomal/nuclear UnCAs in lymphocytes, non-cancerous somatic cells, from patients with HL, at three sampling times, before, during and after treatment with ABVD chemotherapy/radiotherapy, and to compare the frequencies of conventional CA, reported in a previous longitudinal study [7], with chromosomal/nuclear UnCAs, to assess their potential as complementary markers of genomic instability induced in vivo.

## 2. Materials and Methods

### 2.1. Study Population

HL survivors were recruited from the Survivor Clinic of the National Institute of Pediatrics and the National Cancer Institute in Mexico City. A total of five HL patients participated in this study, each providing peripheral blood samples at three time points: before chemotherapy (BT), during chemotherapy (between the third and fourth cycles; DT), and one year after completing treatment (1yAT). At the time of manuscript preparation, all participants were in remission, with no evidence of relapse or secondary malignancies. In addition, a control group of five healthy young individuals (mean age: 23.6 years) was recruited for comparative analysis.

This study was approved by the Ethics Review Board of the National Institute of Pediatrics. All participants provided written informed consent and completed a health and lifestyle questionnaire, which included information on alcohol consumption and smoking habits. To be eligible, the patients had to be diagnosed with Hodgkin’s lymphoma (HL) based on strict clinical and laboratory criteria and selected to receive ABVD chemotherapy and radiotherapy. The HL patients received between 6 and 8 cycles of ABVD. Additionally, the patients underwent field radiotherapy using a Clinac 2100D/C linear accelerator, with doses ranging from 30 to 45 Gray (Gy) of X-rays, delivered over 15 to 30 daily sessions. No participants were excluded from this study, as none reported the use of illicit or pharmaceutical drugs, had received a blood transfusion within six months prior to enrollment, or had a history of relapse or secondary malignancy.

### 2.2. Lymphocyte Culture

Approximately 4 mL of peripheral blood was collected via venipuncture by a certified phlebotomist. The blood samples were cultured for 48 h in RPMI medium (Gibco^TM^, Grand Island, NY, USA), supplemented with phytohemagglutinin (PHA-M; Gibco^TM^, Grand Island, NY, USA) to stimulate lymphocyte proliferation. After culture, colchicine [0.08 µg/mL] (Sigma, St. Louis, MO, USA) was added three hours prior to harvesting. The cells were then exposed to a hypotonic treatment with KCl [0.075 M] (Sigma, St. Louis, MO, USA) for 45 min, followed by washing and fixation using Carnoy’s fixative (methanol/acetic acid, 3:1 Merck& Co. Inc., Rahway, NJ, USA). Slides previously processed for M-FISH and analyzed as described by Ramos et al. [7] were reused by first removing the coverslips and Vectashield mounting medium with three washes in 2X SSC (Sigma, St. Louis, MO, USA). The slides were then dehydrated in graded ethanol (Merck& Co. Inc., Rahway, NJ, USA) solutions (70%, 80%, and 100%) for 2 min each. Finally, the slides were stained using Wright–Giemsa stain (Gibco^TM^, Grand Island, NY, USA), following standard protocols and analyzed for UnCAs using a Zeiss Axiolab 5 microscope at 100× magnification (Zeiss, Oberkochen, Germany).

### 2.3. Cell Analysis

**Figure 1:**
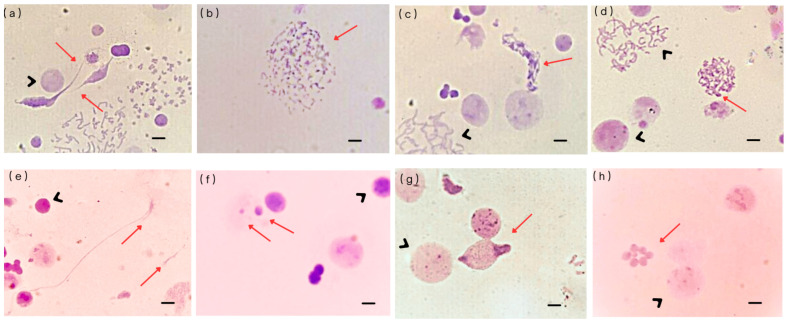
Eight types of UnCA were recorded: (**a**) free chromatin, (**b**) chromosome fragmentation (C-Frag), (**c**) defective mitotic figures (DMFs), (**d**) sticky chromosomes, (**e**) unit fibers, (**f**) micronuclei (MN), (**g**) abnormal nuclear morphology, and (**h**) micronuclei clusters. (―) bar represents a 10 μM scale. Red arrows indicate chromosomal/nuclear UnCA. Black arrowheads indicate normal chromosomes or nuclei.

For each sample, the number and types of UnCAs were recorded, 3105–7760 nuclei were analyzed, and the results were expressed as a percentage of UnCAs. To ensure objectivity, the slides were coded by an independent individual not involved in this study, allowing for blinded analysis. In cases of uncertainty regarding the classification of any UnCA, a second reviewer was consulted to confirm the abnormality type. Following the criteria described by Heng et al. [15], eight distinct types of UnCAs were identified in cultures prepared using conventional cytogenetic methods (including hypotonic treatment, fixation, and air-drying): free chromatin; defective mitotic figures (DMFs); sticky chromosomes; unit fibers; chromosome fragmentation (C-Frag); abnormal nuclear morphology; micronuclei (MN) and MN clusters. These alterations are illustrated in Figure 1.

Definition of Unclassified Chromosomal Alterations [15]:

(a) Free chromatin: These structures consist of interphase chromatin that is not enclosed by a nuclear envelope. They have been observed in various cell lines exposed to chemotherapeutic agents, showing a clear dose–response relationship [12]. Their formation is likely related to nuclear envelope instability, primarily because of underlying genomic instability. Another possible origin of free chromatin is the disruption of micronuclei (MN), which has been identified as one of their possible fates [20].
(b) Defective mitotic figures (DMFs): These structures are characterized by abnormal mitotic figures in which chromosomes and chromatin fibers coexist, indicating incomplete or faulty chromatin condensation. The primary mechanism underlying DMF formation involves defects in chromatin condensation combined with G2–M checkpoint failure. DMFs have been frequently observed in oncological cell lines, supporting the hypothesis that aberrant chromosomal condensation contributes to cancer development.
(c) Sticky chromosomes: These aberrations involve chromosomes that adhere to one another, entangled by chromatin fibers, often appearing as fuzzy or clumped structures. Their formation is linked to abnormal methylation patterns that disrupt proper chromatin condensation. There appears to be a strong association between sticky chromosomes and defective mitotic figure formation. Sticky chromosomes are commonly observed in cells treated with ethidium bromide for high-resolution chromosome preparations, or in cells exposed to agents that interfere with DNA replication, methylation, or chromosomal compaction.
(d) Unit fibers: These are substructures of metaphase chromosomes with a diameter approximately five times smaller than that of a fully condensed chromatid, around 0.4 µm. Unit fibers were first observed by Back [21], who interpreted them as intermediates in chromosome condensation, likely resulting from the coiling of the 250–300 Å solenoid fiber. Unit fibers represent a disruption in normal chromosomal condensation. They have been observed in short-term lymphocyte cultures treated with topoisomerase II inhibitors [15], and their appearance likely reflects incomplete chromatid condensation during metaphase, as result of genotoxic treatment.
(e) Chromosome fragmentation (C-Frag): C-Frag is a distinct form of cell death during mitosis, characterized by the progressive degradation of chromosomes. It was previously misidentified as chromatin pulverization or premature chromosome condensation. However, unlike apoptosis or mitotic catastrophe, C-Frag arises in response to various cellular stressors, such as gene mutations, infections, drug treatments, and centrosomal dysfunction, and is now recognized as a general stress response mechanism. Some studies suggest that C-Frag may lead to aneuploidy; when it affects a single chromosome, it can trigger genomic chaos, including chromothripsis, ultimately contributing to karyotypic abnormalities [22].
(f) Micronuclei (MN): These are small nuclei containing one or a few whole chromosomes, chromosomal fragments, or both. They form through various mechanisms, including chromosome displacement during metaphase, slow chromosome separation during transitions from anaphase to telophase, both bipolar and multipolar, fragments from broken chromosome bridges, inheritance of MN from mother cells, separated nuclear fragments during anaphase, extrusion of chromosomes into a mini cell that fuses with a daughter cell, and formation of nuclear buds during the interphase. MN formation is linked to dysfunction in gene networks responsible for DNA damage response, commonly observed in cancers.

It has been shown that the frequency of MN in peripheral blood lymphocytes is elevated after environmental exposure, such as to harmful chemicals and radiation, and its presence is related to genomic instability [15] and pathophysiological conditions such as DNA double-strand breaks, altered DNA repair response, inappropriate DNA replication, treatment of DNA with adduct-forming chemicals, inhibition of microtubule polymerization, and centromere interference [23,24]. It has been suggested that MNs not only indicate genotoxic effects and genomic instability, but also cellular stress response [25] and immune activity followed by DNA damage [26,27,28]. This makes MN valuable biomarkers in genetic toxicology and cancer research [29].

(g) Micronuclei cluster: This is a group of micronuclei of varying sizes that typically form within a single cell, which can be either diploid or polyploid. These clusters can evolve over time through processes of cell fusion and fission, acting as transitional structures during cancer macroevolution (large-scale genomic changes driving cancer progression) [30]. Their formation depends on both the intensity and duration of stress or damaging exposure on cells, which triggers diverse cellular responses, including activation of apoptotic pathways. The formation and evolution of micronuclei clusters may be connected to genomic chaos, reflecting massive genomic rearrangements and instability [23,30]. It has been proposed that MN clusters arise as a consequence of frustrated cell death. These clusters of MN undergo repeated cycles of fusion and fission, reflecting a trial-and-error process in the search for viable genomic configurations. Through these dynamic events, the genomic content is rearranged, leading to the generation of novel, unstable genomes a phenomenon known as genomic chaos. While most MN clusters do not persist, a small subset may survive and initiate macroevolutionary changes that contribute to cancer development [22,23].
(h) Abnormal nuclear morphology: It is characterized by markedly altered chromatin condensation patterns within the nucleus and it exhibits a dose-dependent response to chemotherapeutic agents such as doxorubicin. These nuclear changes reflect underlying damage to DNA and disruptions in nuclear organization, often linked to the cytotoxicity of chemotherapy [15].

### 2.4. Statistical Analysis

Differences in the proportions of UnCAs between HL patients and healthy donors were evaluated using the Chi-squared with Yates’ correction test when comparing two groups, whereas the Kruskal–Wallis and Wilcoxon test were used to compare all groups. Statistical significance was set at *p* < 0.05.

## 3. Results

### 3.1. Patients

Table 1 presents the clinical history of the HL group, including BT, DT, and 1yAT. The average age of the survivors was 25.2 years, with ages at diagnosis ranging from 18 to 33 years.

### 3.2. Unclassified Chromosomal Aberrations (UnCAs)

#### 3.2.1. UnCAs in Hodgkin’s Lymphoma Patients Versus Healthy Individuals

**Figure 2:**
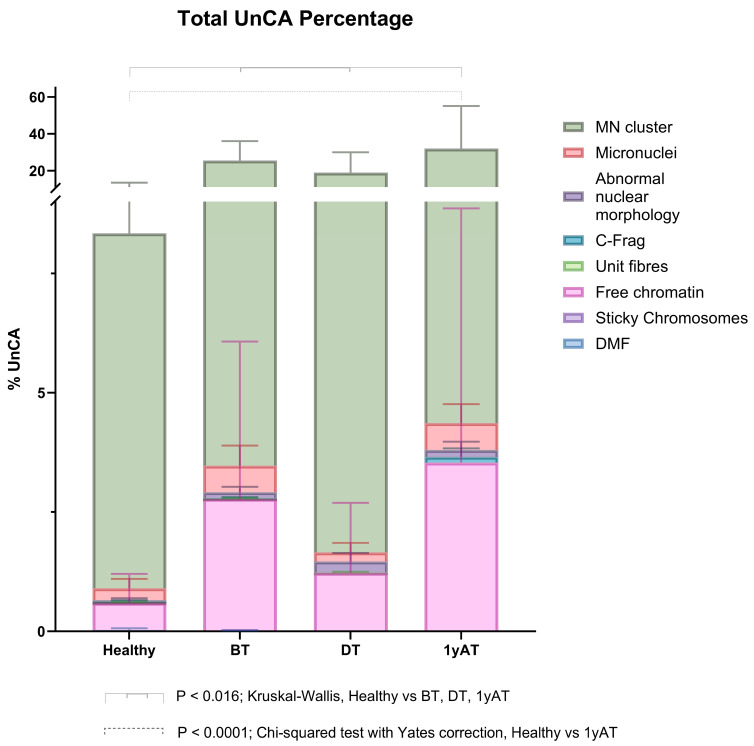
Total percentages of UnCAs observed in the studied population. *p* < 0.0001; Chi-squared test, Yates’ correction. Healthy individuals vs. HL patient’s sampling time (before treatment BT, during treatment DT, and one year after treatment 1yAT). BT vs. 1yAT, BT vs. DT, DT vs. 1yAT *p* < 0.0001; Chi-squared test, Yates’ correction. *p* = 0.016; Kruskal–Wallis test Healthy vs. HL.

UnCAs were observed in all analyzed samples, including those from healthy individuals (Table 2). In the healthy group, the percentage of abnormal cells was 8.16. In HL patients, the total percentage of UnCAs was highest in the 1yAT samples, compared to BT and during-treatment, DT, samples. Specifically, the UnCA percentages were 30.62% for 1yAT, 23.92% for BT, and 18.58% for DT. These values were significantly higher than those observed in healthy individuals (*p* < 0.016; Kruskal–Wallis test) (Table 2, Figure 2).

**Figure 3:**
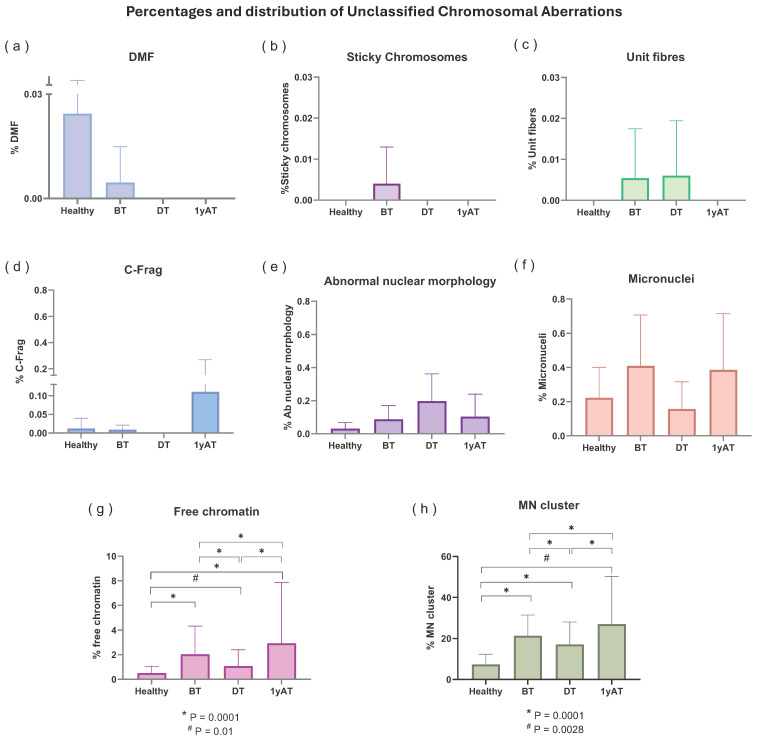
Percentage of each type of unclassified chromosomal aberrations in the study population. To facilitate comparison among the various chromosomal and nuclear alterations, UnCAs with comparable frequencies were systematically grouped, and corresponding scales were constructed for analysis. (**a**) DMF: defective mitotic figures; (**b**) sticky chromosomes; (**c**) unit fibers; (**d**) C-Frag: chromosome fragmentation; (**e**) Abnormal nuclear morphology; (**f**) micronuclei; (**g**) free chromatin; (**h**) MN cluster: micronuclei cluster. Significant differences were observed in the frequencies of free chromatin and micronucleus MN clusters, as determined by the Chi-squared test with Yates’ correction.

Among the eight types of UnCA, MN clusters were the most common, representing approximately 90% of the nuclear abnormalities in HL patients and healthy individuals (Table 2, Figure 3); there was at least a 2.3-fold increase in the percentage of MN clusters in all HL patients as compared with healthy individuals. The highest fold increase in MN cluster (3.7) was observed in HL samples collected one-year AT. All values were significantly different (*p* < 0.013; Kruskal–Wallis test) compared to the values of the MN cluster observed in the healthy individual samples.

**Figure 4:**
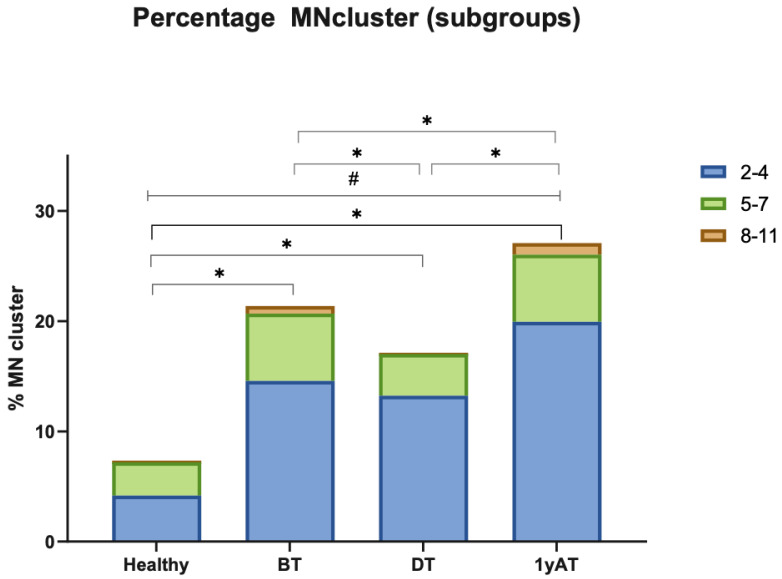
MN clusters were classified into three subgroups according to the number of MN per cluster: 2–4, 5–7, and 8–11. * *p* < 0.0001 Chi-squared with Yates’ correction, Healthy vs. HL each sampling time, (before treatment BT, during treatment DT, and one year after treatment 1yAT). * *p* < 0.0001 Chi-squared with Yates’ correction BT vs. 1yAT, BT vs. DT, DT vs. 1yAT. ^#^ *p*= 0.013; Kruskal–Wallis, Healthy vs. HL (BT, DT, 1yAT). Comparisons were made with number of events per 3000 nuclei.

We classified MN clusters into three subgroups depending on the number of MN observed: 2–4, 5–7, and 8–11 (Figure 4). In all HL samples, the predominant MN clusters were composed of two to four MN, regardless of the time of sampling.

Free chromatin was the second most common type of UnCAs, accounting for 5–8% of all UnCAs observed across all sample groups, including healthy individuals. Although the difference was not statistically significant, a higher frequency of this UnCA was noted in patient samples (Table 2, Figure 3). Overall, UnCAs in the 1yAT samples showed a significantly increased frequency compared to those in healthy individuals, as well as in the BT and DT samples.

All other types of UnCAs were observed at low frequencies and without significantly differences among sampling time or studied group of individuals. An analysis of these low-frequency UnCAs showed that MN presented with the same pattern of free chromatin and MN clusters although without statistical significance. Also, a non-significant increase in abnormal nuclear morphology was noted in the DT patient group and chromosome fragmentation (C-Frag) frequency was more frequent in samples collected 1yAT, without a statistically significant difference (Figure 3).

#### 3.2.2. UnCA vs. Chromosomal Aberrations

**Figure 5:**
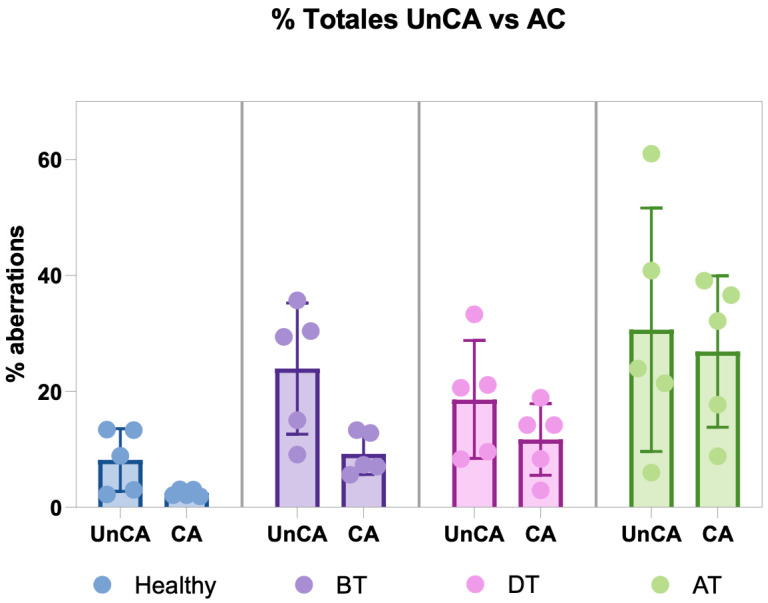
Comparison of the percentages of cells with unclassified chromosomal/nuclear aberrations (UnCAs) and cells with classical chromosomal aberrations (CA) across study groups, including healthy individuals and HL patients at three sampling time points (before treatment BT, during treatment DT, and one year after treatment 1yAT). The percentage of abnormal cells within each group was compared using the Wilcoxon test, and no statistically significant differences were identified.

The comparison between UnCA and classical chromosomal aberrations, CAs, was based on the number of aberrant cells, as this is the only feasible standardization for both analyses. This approach was necessary because UnCA quantifies the abnormal cells, whereas CA analysis is traditionally reported as the frequency of aberrations per cell, though it can also be expressed as the percentage of aberrant cells. When comparing the total number of cells with UnCA across all study groups, including healthy individuals and HL patients at three different sampling time points, a parallel pattern emerged, mirroring the trends observed in the CA analyses. Consistent with previous CA findings, a lower frequency of CA and chromosomal/nuclear UnCAs was detected in DT versus AT, indicating that the most severely damaged cells may be eliminated by chemotherapy and therefore underrepresented in cytogenetic assessments [7]. Interestingly, the aberrant cells, both with UnCAs and CA showed the highest frequency recorded one year after treatment; both analytical methods corroborated this trend, suggesting a temporal pattern in the occurrence of genomic aberrations and reflecting the dynamic nature of genomic instability across different stages of treatment and recovery in HL patients (Figure 5, Table 3).

## 4. Discussion

In this study, we investigated the quantity and types of chromosomal/nuclear UnCAs in lymphocytes from HL patients, assessed before, during, and after anticancer treatment, to evaluate whether these underreported alterations are induced by radio/chemotherapy, and to determine whether their occurrence parallels that of classical CAs, which are commonly used to assess the genotoxic effects of such treatments. Our results showed that when comparing the total number of cells with UnCAs across all study groups, including healthy individuals and HL patients at three different sampling times, the emerging pattern mirrors the trends observed with the classical CA analyses. These results suggest that anticancer-therapy-induced stress leads to chromosomal and nuclear alterations, evident in different phases of the cell cycle. Furthermore, chromosomal/nuclear UnCAs, similar to classical CAs, may serve as valuable indicators for detecting chromosomal instability (CIN) resulting from exposure to genotoxic agents. These genotoxic-stress-induced NCCAs lead to genomic heterogeneity in the hematopoietic stem cells, the essential factor for macroevolution toward cancers, and may partially explain the development of secondary new blood cancers in HL survivors.

As regards the basal frequencies of chromosomal/nuclear UnCA, we found a percentage of 8.16 total UnCAs in healthy individuals, revealing that the most represented type is MN clusters, with a mean of percentage of 7.36, and showing extremely low percentages of the other types of UnCAs.

Chromosomal/nuclear UnCAs showed a higher level of genomic damage in patients with HL compared to healthy individuals, even prior to the initiation of treatment. This observation suggests that the disease itself, as in other cancers [31,32], may be inherently associated with increased genomic instability in HL patient’s normal lymphocytes and this is independent of Hodgkin’s and Reed–Sternberg cancer cells in HL, which are known to undergo extensive genomic modifications [33,34].

When UnCA levels were analyzed at the DT and 1yAT time points, we found that despite the therapeutic success in eliminating cancer cells, the total number of UnCAs was significantly higher in lymphocytes from HL survivors one year after treatment (1yAT). This finding underscores the persistence of damaged cells with a relative survival advantage (“better fitness”), which may sustain the substantial high burden of treatment-induced genomic damage in the patients’ non-cancerous cells.

In addition, we compared the analysis of UnCAs with that of classical CAs as a response to anticancer therapy in patients with HL. This comparison was motivated by previous findings with classical CAs analysis, which showed a low proportion of genomic damage occurring DT, a period when cells are directly exposed to genotoxic agents [7]. We aimed to investigate whether substantial genomic damage might be detectable at other stages of the cell cycle, beyond mitosis. Interestingly, the percentages of aberrant cells obtained from both analyses revealed parallel trends. In both cases, a lower level of genotoxic damage was observed DT as compared with the levels detected 1yAT. This may be attributed to the high rate of cell death induced by chemotherapy, which eliminates the most severely damaged cells, rendering them undetectable by either method. In contrast, in the 1yAT phase, surviving cells accumulate additional alterations, reflecting CIN, a driver of cancer evolution [13,14,35]; in this context, previous studies have reported that abnormalities in nuclear architecture, such as UnCAs, are associated with cellular dysfunction, impaired molecular maintenance mechanisms, and, ultimately, chromosomal breaks that contribute to genomic instability [36].

Our results indicate that MN clusters were the most frequently observed UnCAs (Table 2, Figure 2, Figure 3 and Figure 4). Notably, these clusters showed the greatest differences in frequency between healthy individuals and HL patients and across the different sampling times. Specifically, HL patients exhibited a 2.3-fold increase in MN clusters compared to healthy individuals. Among the HL patient groups, the highest increase, a 3.7-fold rise, was observed in samples 1yAT (Figure 3). Indeed, these types of UnCAs were less frequently observed DT, and those detected at this time point typically involved smaller clusters containing only 2–4 micronuclei per cluster, while the clusters with 8–11 MN re-appeared 1yAT (Figure 3). This supports the idea that the most severely damaged genomes are eliminated during therapy, and that surviving cells with CIN undergo genomic rearrangement. This process facilitates the emergence of NCCAs, which may become detectable at later time points. Notably, we observed a resurgence of these 8–11 MN clusters 1yAT. It is hypothesized that MN clusters act as intermediary structures for genomic reorganization, promoting the formation of new genomic architectures such as genomic chaos, conducive to macroevolutionary selection [11]. Interestingly, chromoplexy (a hallmark of genomic chaos) was detected in the analysis with M-FISH of the HL survivors at 1yAT [7]. This temporal coincidence with the peak of MN clusters suggests a possible mechanistic link, positioning MN clusters as a possible precursor of chromoplexy, and genomic chaos as a potential outcome of treatment-induced genomic remodeling.

The genomic heterogeneity induced by anticancer agents in lymphocytes, and possibly in all somatic cells of cancer survivors, is of critical importance, as each altered genome constitutes a distinct information system with a unique potential for survival. If any of these genomic variants acquire a selective growth advantage, they may initiate a transition from punctuated macroevolution to gene-mutation-driven microevolution [11]. This process could ultimately lead to the development of secondary malignancies, either hematologic or solid tumors, a phenomenon well documented among survivors of Hodgkin’s lymphoma (HL) [37].

This information underscores the lasting impact of anticancer therapies on genomic stability and highlights the potential utility of UnCAs as markers for assessing treatment-related genomic damage over time. Chromosomal/nuclear UnCAs analysis revealed genomic alterations within interphase nuclei, thereby complementing the damage detected by classical CA. Further research is warranted to investigate this phenomenon, which remains detectable even in samples collected a decade after treatment, such as those from patients treated with MOPP, as reported by Salas et al. and Bilban-Jakopin [38,39]. These findings suggest that UnCAs are genome-level alterations that may be initiate a macroevolution steps toward cancer and could serve as a valuable tool for monitoring the long-term genomic effects of anticancer therapies, potentially useful for both treatment strategies and follow-up care for cancer survivors.

## 5. Conclusions

An analysis of UnCAs across different treatment stages revealed a higher frequency in HL patients than in healthy individuals, even before treatment initiation, suggesting an inherent genomic instability associated with the disease.

The analyses of UnCAs across all study groups, including healthy individuals and HL patients at three different sampling times, revealed a genomic damage pattern that mirrors the trends observed with the classical CA analyses, showing DT with a low percentage of damaged cells as compared with the proportion of cells with cytogenetic abnormalities in the 1yAT samples.

The sustained presence and even the amplification of UnCAs observed 1yAT in blood cells suggest that hematopoietic stem and progenitor cells that survived treatment were able to replenish the hematopoietic compartment, albeit at the cost of carrying sublethal genomic damage. The cell population that survived the stress induced by ABVD chemotherapy and/or radiotherapy 1yAT exhibits pronounced genomic heterogeneity, a well-established driver of cancer progression. This genomic variability may help explain the elevated risk of secondary malignancies, both hematologic and solid tumors, commonly observed in survivors of HL.

This study also highlights the reliability and advantages of UnCAs analysis as a complementary tool to classical cytogenetic methods, providing an accessible and broadly applicable approach for assessing genomic instability.

## Figures and Tables

**Table 1:** Patient clinical data.

ID Patient		66	111	118	122	109
**Gender**		Male	Male	Female	Male	Male
**Diagnostic age**		18	22	25	28	33
**Subtype HD**		Mixed cellularity	Mixed cellularity	Nodular sclerosis	Mixed cellularity	Mixed cellularity
**Stage**		IIB	IB	IIB	IIB	IIIA
**Radiotherapy**	**Field**	Neck	Neck	Neck and mediastinum	Mediastinum	No data available
	**Dose**	30 Gy	30 Gy	36 Gy	45 Gy	
	**Sessions**	20	10	20	25	
	**Months after chemotherapy**	5	4	6	6	
**ABVD cycles**		8	8	6	6	
**Before treatment**	**Medical assessment at blood sampling**	Leukopenia due to lymphopenia.	Microcytosis. High level of B-globulin.	Macrocytosis. Elevated monocyte levels	Iron deficiency anemia. Lymphopenia possibly due to invasion of cancer cells, and high levels of eosinophils, neutrophils and monocytes.	
**During treatment (between 3rd–4th cycle)**		Leukopenia due to lymphopenia, this sample showed the lowest levels of cellularity in this patient.	Normal lymphocyte levels. Elevated monocyte levels.	Macrocytosis. Leukopenia due to lymphopenia. Elevated monocytes levels.	Anaphylactic reaction to bleomycin with skin toxicity (treated with anti-steroids). Elevated eosinophil counts (commonly associated with allergic clinical features).	
**After treatment**		Leukopenia due to lymphopenia.	Normal leukocyte levels with a slight decrease in mean platelet volume.	Mild lymphopenia. Mild macrocytosis.	Values of hematocrit and monocytes slightly above normal, while eosinophils raised very close to the value of the sample before treatment.	
**Months after radiotherapy/chemotherapy (sample AT)**		12/17	12/16	12/18	9/15	
**Remission**		After finished anticancer treatment, FDG-PET was negative for tumor activity. Without relapse and asymptomatic				

**Table 2:** Percentage of chromosomal/nuclear UnCAs.

Sampling Time	Patient ID	# Studied Cells	DMF	Sticky Chromosomes	Free Chromatin	Unit Fiber	C-Frag	AbNM	MN	MN Cluster	TOTAL UnCA
**Before Treatment**	**66-I**	4295	0.02	0.00	0.95	0.00	0.02	0.00	0.35	28.03	29.40
	**109-I**	3688	0.00	0.00	2.03	0.03	0.00	0.00	0.70	12.30	15.00
	**111-I**	3355	0.00	0.00	0.14	0.00	0.00	0.18	0.12	8.61	9.10
	**118-I**	4762	0.00	0.02	5.96	0.00	0.02	0.14	0.74	28.85	35.70
	**122-I**	4315	0.00	0.00	1.06	0.00	0.00	0.12	0.14	29.04	30.40
**Mean**		4083	0.004	0.004	2.03	0.01	0.01	0.09	0.41	21.37	23.92
**SD**		558.08	0.01	0.01	2.30	0.01	0.01	0.08	0.30	10.05	11.29
**During Treatment**	**66-II**	3903	0.00	0.00	0.10	0.00	0.00	0.13	0.23	20.60	21.10
	**109-II**	3339	0.00	0.00	0.09	0.03	0.00	0.48	0.00	8.99	9.60
	**111-II**	4501	0.00	0.00	0.20	0.00	0.00	0.07	0.00	33.04	33.30
	**118-II**	3778	0.00	0.00	2.09	0.00	0.00	0.11	0.37	18.03	20.60
	**122-II**	3278	0.00	0.00	2.90	0.00	0.00	0.21	0.18	5.00	8.30
**Mean**		3760	0.00	0.00	1.08	0.01	0.00	0.20	0.16	17.13	18.58
**SD**		494.83	0.00	0.00	1.33	0.01	0.00	0.17	0.16	10.95	10.17
**1yAfter Treatment**	**66-III**	3822	0.00	0.00	11.72	0.00	0.21	0.31	0.98	8.16	21.38
	**109-III**	3203	0.00	0.00	0.94	0.00	0.34	0.00	0.31	4.40	5.99
	**111-III**	5092	0.00	0.00	0.10	0.00	0.00	0.04	0.20	40.46	40.79
	**118-III**	7760	0.00	0.00	0.90	0.00	0.00	0.00	0.26	59.86	61.02
	**122-III**	4067	0.00	0.00	0.98	0.00	0.00	0.17	0.20	22.57	23.92
**Mean**		4789	0.00	0.00	2.93	0.00	0.11	0.10	0.39	27.09	30.62
**SD**		1795.16	0.00	0.00	4.93	0.00	0.16	0.14	0.33	23.16	21.00
**Healthy individuals**	**NL-31**	3468	0.06	0.00	0.35	0.00	0.00	0.00	0.38	12.66	13.44
	**NL-32**	3105	0.06	0.00	0.06	0.00	0.00	0.03	0.10	2.71	2.96
	**NL-34**	3464	0.00	0.00	1.18	0.00	0.00	0.03	0.09	12.04	13.34
	**NL-35**	3114	0.00	0.00	0.00	0.00	0.00	0.00	0.10	2.06	2.15
	**NL-36**	3293	0.00	0.00	0.97	0.00	0.061	0.09	0.46	7.32	8.90
**Mean**		3289	0.02	0.00	0.51	0.00	0.012	0.03	0.22	7.36	8.16
**SD**		178.30	0.03	0.00	0.54	0.00	0.027	0.04	0.18	4.99	5.44

DMF = defective mitotic figures; C-Frag = chromosome fragmentation; AbNM = abnormal nuclear morphology; MN = micronuclei; MN cluster = micronuclei cluster.

**Table 3:** Percentage of cells with unclassified chromosomal/nuclear aberrations (UnCAs) vs. percentage of cells with classical chromosomal aberrations (CAs).

Healthy					Hodgkin’s Patients												
**NL** **ID**	Healthy				ID	BT				DT				AT			
	Abnormal Metaphases	Abnormal Nuclei	UnCA	CA		Abnormal Metaphases	Abnormal Nuclei	UnCA	CA	Abnormal Metaphases	Abnormal Nuclei	UnCA	CA	Abnormal Metaphases	Abnormal Nuclei	UnCA	CA
31	0.06	13.3	13.4	2.1	66	0.05	29.3	29.4	5.6	0.00	21.1	21.1	2.9	0.20	21.2	21.4	17.7
32	0.06	2.9	3	3.03	109	0.03	15.0	15	12.8	0.03	9.5	9.6	18.9	0.3	5.6	6	36.58
34	0.00	13.3	13.3	1.9	111	0.00	9.1	9.1	7.31	0.00	33.3	33.3	14.2	0.00	40.8	40.8	8.82
35	0.00	2.15	2.2	2.1	118	0.04	35.7	35.7	13.3	0.00	20.6	20.6	14.2	0.00	61.0	61	32.14
36	0.06	8.84	8.9	3.1	122	0.00	30.4	30.4	7.04	0.00	8.3	8.3	8.33	0.00	23.9	23.9	39.1
Mean ± SD	0.04 ± 0.03	8.12 ± 5.44	8.2 ± 5.4	2.4 ± 0.57	Mean ± SD	0.02 ± 0.02	23.8 ± 11.2	23.92 ± 11.29	9.21 ± 3.57	0.005 ± 0.01	18.5 ± 10.1	18.58 ± 10.17	11.7 ± 6.19	0.11 ± 0.15	30.5 ± 21.1	30.62 ± 20.99	26.87 ± 13.05

Number of metaphases analyzed in CA: NL31.-95; NL32.-99; NL34.-107; NL35.-93; NL36.-95. BT66.-54; BT109.-70; BT111.-82; BT118.-75; BT122.-71. DT66.-68; DT109.-79; DT111.-63; DT118.-98; DT122.-84. AT66.-62; AT109.-82; AT111.-102; AT118.-56; AT122.-64 [7].

## Data Availability

The data presented in this study are available in this article.

## Abbreviations

The following abbreviations are used in this manuscript:

HL	Hodgkin’s lymphoma
BT	Before treatment
DT	During treatment
AT	After treatment
1yAT	One year after treatment
ABVD	Adriamycin, Bleomycin, Vinblastine, Dacarbazine
UnCA	Unclassified chromosomal aberrations
NCCA	Non-clonal chromosomal aberrations
SMN	Second malignant neoplasm
DMFs	Defective mitotic figures
C-Frag	Chromosome fragmentation
MN	Micronuclei
MN cluster	Micronuclei cluster
Gy	Gray
M-FISH	Multiplex fluorescence in situ hybridization
SSC	Saline sodium citrate
SD	Standard deviation
CA	Chromosomal aberrations
MOPP	Mechlorethamine, Oncovine, Procarbazine, Prednisone

## References

[1] Kaatsch P., Byrne J., Grabow D. (2021). Managing a Pan-European Consortium on Late Effects among Long-Term Survivors of Childhood and Adolescent Cancer—The Pancarelife Project. Int. J. Environ. Res. Public. Health.

[2] Connors J.M., Cozen W., Steidl C., Carbone A., Hoppe R.T., Flechtner H.H., Bartlett N.L. (2020). Hodgkin Lymphoma. Nat. Rev. Dis. Primers.

[3] Sung H., Ferlay J., Siegel R.L., Laversanne M., Soerjomataram I., Jemal A., Bray F. (2021). Global Cancer Statistics 2020: GLOBOCAN Estimates of Incidence and Mortality Worldwide for 36 Cancers in 185 Countries. CA Cancer J. Clin..

[4] Zahnreich S., Schmidberger H. (2021). Childhood Cancer: Occurrence, Treatment and Risk of Second Primary Malignancies. Cancers.

[5] Farnetani G., Vannucci M., Fino M.G., Cioppi F., Rosta V., Palma M., Tamburrino L., Vinci S., Casamonti E., Degl’Innocenti S. (2024). Severe Sperm DNA Fragmentation May Persist for up to 3 Years after Cytotoxic Therapy in Patients Affected by Hodgkin Lymphoma and Non-Hodgkin Lymphoma. Hum. Reprod..

[6] Engert A., Younes A. (2015). Hodgkin Lymphoma: A Comprehensive Update on Diagnostics and Clinics.

[7] Ramos S., Navarrete-Meneses P., Molina B., Cervantes-Barragán D.E., Lozano V., Gallardo E., Marchetti F., Frias S. (2018). Genomic Chaos in Peripheral Blood Lymphocytes of Hodgkin’s Lymphoma Patients One Year After ABVD Chemotherapy/Radiotherapy. Environ. Mol. Mutagen..

[8] Frias S., Ramos S., Salas C., Molina B., Sánchez S., Rivera-Luna R. (2019). Nonclonal Chromosome Aberrations and Genome Chaos in Somatic and Germ Cells from Patients and Survivors of Hodgkin Lymphoma. Genes.

[9] Ye C.J., Sharpe Z., Heng H.H. (2020). Origins and Consequences of Chromosomal Instability: From Cellular Adaptation to Genome Chaos-mediated System Survival. Genes.

[10] De Vries S., Schaapveld M., Janus C.P.M., Daniëls L.A., Petersen E.J., Van Der Maazen R.W.M., Zijlstra J.M., Beijert M., Nijziel M.R., Verschueren K.M.S. (2021). Long-Term Cause-Specific Mortality in Hodgkin Lymphoma Patients. J. Natl. Cancer Inst..

[11] Heng E., Thanedar S., Henrg H.H. (2024). The Importance of Monitoring Non-Clonal Chromosome Aberrations (NCCAs) in Cancer Research. Methods Mol. Biol..

[12] Liu G., Stevens J.B., Horne S.D., Abdallah B.Y., Ye K.J., Bremer S.W., Ye C.J., Chen D.J., Heng H.H. (2014). Genome Chaos: Survival Strategy during Crisis. Cell Cycle.

[13] Heng H.H.Q., Bremer S.W., Stevens J., Ye K.J., Miller F., Liu G., Ye C.J. (2006). Cancer Progression by Non-Clonal Chromosome Aberrations. J. Cell. Biochem..

[14] Heng H.H.Q., Stevens J.B., Liu G., Bremer S.W., Ye K.J., Reddy P.V., Wu G.S., Wang Y.A., Tainsky M.A., Ye C.J. (2006). Stochastic Cancer Progression Driven by Non-Clonal Chromosome Aberrations. J. Cell Physiol..

[15] Heng H.H., Liu G., Stevens J.B., Abdallah B.Y., Horne S.D., Ye K.J., Bremer S.W., Chowdhury S.K., Ye C.J. (2013). Karyotype Heterogeneity and Unclassified Chromosomal Abnormalities. Cytogenet. Genome Res..

[16] Rangel N., Forero-Castro M., Rondon-Lagos M. (2017). New Insights in the Cytogenetic Practice: Karyotypic Chaos, Non-Clonal Chromosomal Alterations and Chromosomal Instability in Human Cancer and Therapy Response. Genes.

[17] Heng J., Heng H.H. (2022). Genome Chaos: Creating New Genomic Information Essential for Cancer Macroevolution. Semin. Cancer Biol..

[18] Fenech M., Kirsch-Volders M., Natarajan A.T., Surralles J., Crott J.W., Parry J., Norppa H., Eastmond D.A., Tucker J.D., Thomas P. (2011). Molecular Mechanisms of Micronucleus, Nucleoplasmic Bridge and Nuclear Bud Formation in Mammalian and Human Cells. Mutagenesis.

[19] Fenech M., Knasmueller S., Bolognesi C., Bonassi S., Holland N., Migliore L., Palitti F., Natarajan A.T., Kirsch-Volders M. (2016). Molecular Mechanisms by Which in Vivo Exposure to Exogenous Chemical Genotoxic Agents Can Lead to Micronucleus Formation in Lymphocytes in Vivo and Ex Vivo in Humans. Mutat. Res. Rev. Mutat. Res..

[20] Guo X., Ni J., Liang Z., Xue J., Fenech M.F., Wang X. (2019). The Molecular Origins and Pathophysiological Consequences of Micronuclei: New Insights into an Age-Old Problem. Mutat. Res. Rev. Mutat. Res..

[21] Bak A.L., Bak P., Zeuthen J. (1979). Higher Levels of Organization in Chromosomes. J. Theor. Biol..

[22] Stevens J.B., Horne S.D., Abdallah B.Y., Ye C.J., Heng H.H. (2013). Chromosomal Instability and Transcriptome Dynamics in Cancer. Cancer Metastasis Rev..

[23] Ye C.J., Sharpe Z., Alemara S., Mackenzie S., Liu G., Abdallah B., Horne S., Regan S., Heng H.H. (2019). Micronuclei and Genome Chaos: Changing the System Inheritance. Genes.

[24] Guo X., Dai X., Zhou T., Wang H., Ni J., Xue J., Wang X. (2020). Mosaic Loss of Human Y Chromosome: What, How and Why. Hum. Genet..

[25] Guo X., Dai X., Wu X., Zhou T., Ni J., Xue J., Wang X. (2020). Understanding the Birth of Rupture-Prone and Irreparable Micronuclei. Chromosoma.

[26] Chatterjee A., Rodger E.J., Eccles M.R. (2018). Epigenetic Drivers of Tumourigenesis and Cancer Metastasis. Semin. Cancer Biol..

[27] Luzhna L., Kathiria P., Kovalchuk O. (2013). Micronuclei in Genotoxicity Assessment: From Genetics to Epigenetics and Beyond. Front. Genet..

[28] Horne S.D., Chowdhury S.K., Heng H.H.Q. (2014). Stress, Genomic Adaptation, and the Evolutionary Trade-Off. Front. Genet..

[29] Fenech M. (2020). Cytokinesis-Block Micronucleus Cytome Assay Evolution into a More Comprehensive Method to Measure Chromosomal Instability. Genes.

[30] Heng E., Moy A., Liu G., Heng H.H., Zhang K. (2021). ER Stress and Micronuclei Cluster: Stress Response Contributes to Genome Chaos in Cancer. Front. Cell Dev. Biol..

[31] Santos R.A., Teixeira A.C., Mayorano M.B., Carrara H.H.A., Andrade J.M., Takahashi C.S. (2010). Basal Levels of DNA Damage Detected by Micronuclei and Comet Assays in Untreated Breast Cancer Patients and Healthy Women. Clin. Exp. Med..

[32] Pelevina I.I., Aleshchenko A.V., Antoshchina M.M., Vorob’eva N.I., Kudriashova O.V., Lashkova O.E., Lizunova E.I., Osipov A.N., Riabchenko N.I., Serebrianyĭ A.M. (2012). Molecular-biological properties of blood lymphocytes of Hodgkin’s lymphoma patients. Plausible possibility of treatment effect prognosis. Radiats Biol. Radioecol..

[33] Falzetti D., Crescenzi B., Matteucci C., Falini B., Martelli M.F., den Berghe M., Mecucci C. (1999). Genomic Instability and Recurrent Breakpoints Are Main Cytogenetic Findings in Hodgkin’s Disease. Haematologica.

[34] Montgomery N.D., Coward W.B., Johnson S., Yuan J., Gulley M.L., Mathews S.P., Kaiser-Rogers K., Rao K.W., Sanger W.G., Sanmann J.N. (2016). Karyotypic Abnormalities Associated with Epstein-Barr Virus Status in Classical Hodgkin Lymphoma. Cancer Genet..

[35] Tijhuis A.E., Foijer F. (2024). Characterizing Chromosomal Instability-Driven Cancer Evolution and Cell Fitness at a Glance. J. Cell Sci..

[36] Pentzold C., Kokal M., Pentzold S., Weise A. (2021). Sites of Chromosomal Instability in the Context of Nuclear Architecture and Function. Cell. Mol. Life Sci..

[37] Kumar V., Garg M., Chandra A.B., Mayorga V.S., Ahmed S., Ailawadhi S. (2018). Trends in the Risks of Secondary Cancers in Patients With Hodgkin Lymphoma. Clin. Lymphoma Myeloma Leuk..

[38] Salas C., Perez-Vera P., Frias S. (2011). Genetic Abnormalities in Leukemia Secondary to Treatment in Patients with Hodgkin’s Disease. Rev. De Investig. Clin.-Clin. Transl. Investig..

[39] Bilban-Jakopin C., Bilban M. (2001). Genotoxic Effects of Radiotherapy and Chemotherapy on Circulating Lymphocytes in Patients with Hodgkin’s Disease. Mutat. Res.-Genet. Toxicol. Environ. Mutagen..

